# Offshore tethered platform springing response statistics

**DOI:** 10.1038/s41598-022-25806-x

**Published:** 2022-12-07

**Authors:** Oleg Gaidai, Jingxiang Xu, Qingsong Hu, Yihan Xing, Fuxi Zhang

**Affiliations:** 1 Shanghai Ocean University, Shanghai, China; 2 University of Stavanger, Stavanger, Norway

**Keywords:** Energy science and technology, Engineering, Mathematics and computing

## Abstract

This paper demonstrates the validity of the Naess–Gadai method for extrapolating extreme value statistics of second-order Volterra series processes through application on a representative model of a deep water small size tension leg platform (TLP), with specific focus on wave sum frequency effects affecting restrained modes: heave, roll and pitch. The wave loading was estimated from a second order diffraction code WAMIT, and the stochastic TLP structural response in a random sea state was calculated exactly using Volterra series representation of the TLP corner vertical displacement, chosen as a response process. Although the wave loading was assumed to be a second order (non-linear) process, the dynamic system was modelled as a linear damped mass-spring system. Next, the mean up-crossing rate based extrapolation method (Naess–Gaidai method) was applied to calculate response levels at low probability levels. Since exact solution was available via Volterra series representation, both predictions were compared in this study, namely the exact Volterra and the approximate one. The latter gave a consistent way to estimate efficiency and accuracy of Naess–Gaidai extrapolation method. Therefore the main goal of this study was to validate Naess–Gaidai extrapolation method by available analytical-based exact solution. Moreover, this paper highlights limitations of mean up-crossing rate based extrapolation methods for the case of narrow band effects, such as clustering, typically included in the springing type of response.

## Introduction

The TLP response is characterized by the distinctive difference in response behavior between the restrained and the compliant degrees of freedom. The compliant degrees of freedom (surge, sway and yaw) exhibit relatively large motion amplitudes and long resonance periods of the order of a 100 s or longer, while the restrained degrees of freedom (heave, roll and pitch) have much sorter resonance periods typically between 2 and 8 s. One of the TLP design objectives is to keep the high frequency response resonance periods (for example of heave or pitch) outside the wave frequency range. However, in deep water, this may not be easily achievable. For the TLP structure studied in this paper, the resonant heave and pitch responses have been influenced by the wave loads generated by combination of the first order and the second order (i.e. sum) frequencies, see^1–14^.

This paper is primarily studying the second order, sum frequency wave loading affecting TLP heave and pitch response. Those latter structural responses are of practical engineering interest since this phenomenon influences TLP tethers structural integrity, and thus has significant effect on the overall TLP structural safety. In other words, the major design challanges related to TLP tethers could be:

- Extreme wave loading of tethers (overload, slack and snap)
- Tether fatigue accumulation and shortening of fatigue life

**Figure 1:**
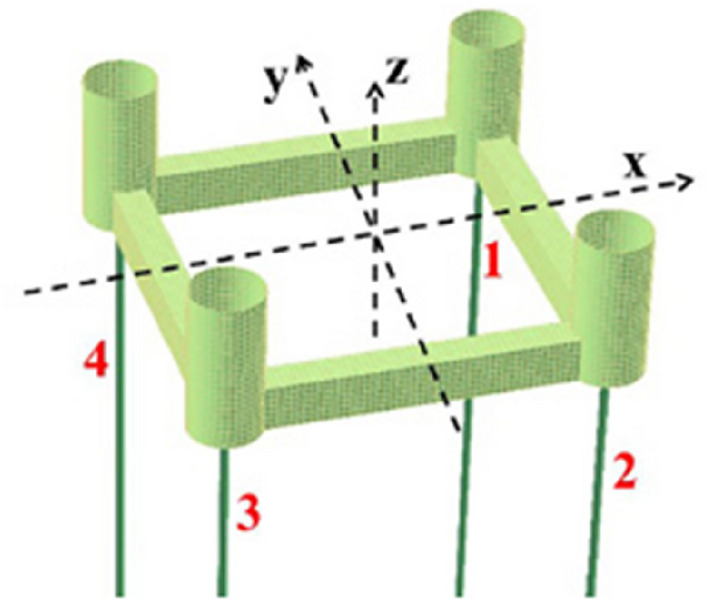
Configuration of TLP and tendons^33^. Distance between each pair of neighbouring tendons is 76 m. Each column radius 12.5 m.

It is important to distinguish between “ringing” and “springing” types of wave loads; an accurate definition is not easily available in the literature. The “ringing” is generally caused by extreme, transient wave loads, resulting in a higher response frequency, which is dangerously near to the resonant structural frequency, see^15^ for the gravity based structure (GBS) ringing study. The “springing” is typically more stationary phenomenon, caused by continuous action of the second order, sum frequency wave load effects as well as by the first order wave load high frequency parts, see e.g.^1,14^. Both ringing and springing may lead to a TLP tether failure. Higher order response components are not negligible when studying extreme responses, as each TLP column has large diameter of 25 m., see Fig. 1. For details see FNV theory^16^ for calculation of higher-order wave loads in deep water, on a vertical, free-surface piercing cylinder.

As mentioned above, the ringing is in fact a rare extreme event, and may contribute to a significant reduction of tether life time in relatively few stress cycles. On the opposite, the springing phenomenon contributes to the long term fatigue damage, that comes from virtually all sea states, see^17,18^.

It is still being debated in the literature, whether the most important forcing mechanism that generates ringing is based on the second, third, fourth or even infinite order wave load effects, see e.g.^19,20^. The latter makes the ringing phenomenon quite challenging to study. In engineering practice, however, it is generally accepted that the critical source of excitation due to springing comes from the sum frequency excitation. However, as already pointed out, the first order loading may also be of importance for springing, especially in harsh sea states with low wave peak periods. There is a number of challenges, related to the accurate springing response calculation—for example, accurate estimation of the TLP damping, which is quite small.

This paper studies the first and second order springing forces and motions at both extreme and moderate response levels. Not much attention has been given in this paper to study non-linear damping effects related to the high frequency heave and pitch motions, however the linear damping values used in the current analysis are considered to be representative, as being consistent with model test data and full scale observations^1^. The tether fatigue life time predictions is a matter of practical engineering interest, however it has been left beyond the scope of the present paper, as the main focus was on the structural extreme response. The origin of the coordinate system is located at the still water level with the positive *z*-axis pointing upwards. In^21^ the operating Heidrun TLP (Norwegian sea) model tests were studied, and tendon tension was analysed.

## Methods

The TLP response process $Z\left( t \right)$ considered here is modelled as a stationary stochastic Volterra series. More specifically, in this paper the TLP heave and pitch motions in random waves have been combined into the vertical TLP corner displacement $Z\left( t \right)$, that has been chosen for the extreme response assessment. This type of response is often approximated with a sum of linear (first order) and second order (sum-frequency springing) component, while higher order terms have been neglected. If the structural springing resonance frequency is located beyond the relevent wave energy frequency range, the first order term can be neglected. Under the latter assumptions, the response $Z\left( t \right)$ can be expressed as follows, see^1,22^

1 $$Z\left( t \right) = \alpha \tilde{W}\left( t \right) + \mathop \sum \limits_{{j = - \tilde{N}}}^{{\tilde{N}}} \left\{ {\beta_{j} W_{j} \left( t \right) + \mu_{j} W_{j}^{2} \left( t \right)} \right\}$$

where $\mu_{ - j} = - \mu_{j}$, $W_{ - j} \left( t \right) = H\left[ {W_{j} \left( t \right)} \right]$, $\alpha$ , $\beta_{j}$ being proper linearization coefficients, with $H$ being Hilbert transform, see^1^. Here $2N$ being the number of two-sided equidistantly discretized wave energy frequency range $\omega_{ - N} < .. < \omega_{ - 1} < 0 < \omega_{1} < .. < \omega_{N} ; \omega_{ - j} = - \omega_{j}$; and $\tilde{W}$, $W_{j}$ are real stationary Gaussian $N\left( {0,1} \right)$-processes. Gaussian process $\tilde{W}$ is uncorrelated with $\left\{ {W_{j} } \right\}_{{j = - \tilde{N}}}^{{\tilde{N}}}$, with $\tilde{N}$ typically being much less than $N$ (see^1^) and coefficients $\mu_{j}$ are obtained by solving the following eigenvalue problem

2 $$\underline{\underline{Q}} \underline {u}_{j} = \mu_{j} \underline {u}_{j}$$

of the Hermitian matrix $\underline{\underline{Q}} = \left( {Q_{kl} } \right)$, with real eigenvalues $\mu_{j}$ and the set of orthonormal eigenvectors $\underline {u}_{j}$, $j = - N, \ldots ,N$ and

3 $$Q_{kl} = \hat{H}_{2} \left( {\omega_{k} , - \omega_{l} } \right) \cdot \frac{1}{2}\left[ {S_{\eta } \left( {\left| {\omega_{k} } \right|} \right)S_{\eta } \left( {\left| {\omega_{l} } \right|} \right)} \right]^{1/2} \Delta \omega$$

where $S_{\eta } \left( \omega \right)$ being the one-sided power spectral density (PSD) of incident waves; while $\hat{H}_{2} \left( {\omega_{k} , - \omega_{l} } \right)$ being the QTF (quadratic transfer function) between the waves and the related vertical displacement (affected by heave and pitch). Two regular wave frequency trains $\left| {\omega_{k} } \right|$ and $\left| {\omega_{l} } \right|$ are being input to a difference-frequency term $\hat{H}_{2} \left( {\left| {\omega_{k} } \right|, - \left| {\omega_{l} } \right|} \right)$ and a sum-frequency term $\hat{H}_{2} \left( {\left| {\omega_{k} } \right|,\left| {\omega_{l} } \right|} \right)$. For the vertical response modes, the difference frequency effects are damped down by dynamics of the TLP structure, see^22^. Thus for the heave and pitch response of a TLP the difference frequency terms can be neglected, leaving only the sum frequency terms. The latter approximation then transforms the $2N \times 2N$ matrix $\underline{\underline{Q}} \user2{ }$ into the following Hermitian form

4 $$\underline{\underline{Q}} = \left( {\begin{array}{*{20}c} 0 & {\underline{\underline{S}}^{H} } \\ {\underline{\underline{S}} } & 0 \\ \end{array} } \right)$$

with $\underline{\underline{S}}^{H} = \left( {\underline{\underline{S}}^{*} } \right)^{T}$ being the Hermitian conjugate of $\underline{\underline{S}}$ matrix, so that the matrix $\underline{\underline{Q}}$ becomes Hermitian. Next, stochastic processes $W_{j} \left( t \right)$ can be decomposed as follows

5 $$W_{j} \left( t \right) = \mathop \sum \limits_{k = - N}^{N} \underline {u}_{j} \left( {\omega_{k} } \right)B_{k} e^{{i\omega_{k} t}}$$

where $\underline {u}_{j} \left( {\omega_{k} } \right)$ being the *k*-th component of $\underline {u}_{j}$ vector. The set $\left\{ {B_{j} } \right\}_{j = - N}^{N}$ consists of complex Gaussian variables $B_{j} = R_{j} + iI_{j}$ such that $\left\{ {R_{j} ,I_{j} } \right\}_{j = 1}^{N}$ being real independent Gaussian $N\left( {0,\frac{1}{\sqrt 2 }} \right)$ variables and $B_{ - j} = B_{j}^{*}$.

For each fixed time $t$,$\left\{ {W_{j} \left( t \right)} \right\}_{j = - N}^{N}$ becomes an array of independent Gaussian variables. Derivations presented above are analogous to the second-order slow drift approximation; for more details, see^23^. Representation given by Eq. (1) leads to the CPU efficient calculation of the mean up-crossing rate function of the response process $Z\left( t \right)$, see^1,24^. Under the Poisson assumption of independent up-crossings of high response levels, the mean up-crossing rate as a function of the response level is of major importance, see^1,24^. Note that CPU time gain/efficiency was estimated by comparing the extra depth of extrapolated PDF tail on the decimal log scale, with respect to a directly Monte Carlo simulated (non-extrapolated) PDF tail.

### Mean up-crossing rate

The probability of exceeding a given response level of interest is tightly related to the mean level up-crossing rate function, see^25^. Therefore, the latter function is a key parameter in estimating extreme response. In this section the calculating procedure of the mean up-crossing rate is briefly described, for more details see^1,24^.

Let $N_{Z}^{ + } \left( \zeta \right)$ be the rate of up-crossings of the response level $\zeta$ by the response process $Z\left( t \right)$, and let $\nu_{Z}^{ + } \left( \zeta \right) = E\left[ {N_{Z}^{ + } \left( \zeta \right)} \right]$, where $\nu_{Z}^{ + } \left( \zeta \right)$ stands for the mean up-crossing rate of the response level $\zeta$. Next, the following Rice formula is introduced

6 $$\nu_{Z}^{ + } \left( \zeta \right) = \mathop \smallint \limits_{0}^{\infty } \dot{\zeta } p_{{Z\dot{Z}}} \left( {\zeta ,\dot{\zeta }} \right)d\dot{\zeta }$$

where $p_{{Z\dot{Z}}} \left( {\zeta ,\dot{\zeta }} \right)$ being the stationary joint PDF (probability density function) of the response process $Z$ and its time derivative $\dot{Z}$. Estimating the mean up-crossing crossing rate of a stationary stochastic process given by a second order stochastic Volterra series directly from Eq. (6) is quite difficult task due to challenges with estimating the joint PDF $p_{{Z\dot{Z}}} \left( {\zeta ,\dot{\zeta }} \right)$. However, the latter difficulty can be circumvented by applying the characteristic function concept. Inverse transformation from the characteristic function back to the joint PDF can be done by applying a CPU efficient and accurate saddle point method, see^1,24^.

### Extrapolation method

As the number of eigenvalues $N$ grows, so grows the CPU time required for accurate up-crossing rate function calculation. It is therefore of interest to study accurate approximations, as for example Naess–Gaidai method, employed later on in this paper. The exceedance probability given the exposure time ${ }T$, can be approximated by the following commonly used approach

7 $${\text{Prob}}\left( {Z\left( t \right) \ge \zeta ; t \in \left[ {0,T} \right]} \right) \approx 1 - {\text{exp}}\left( { - \nu_{Z}^{ + } \left( \zeta \right)T} \right) \approx \nu_{Z}^{ + } \left( \zeta \right)T \ll 1$$

The idea underlying the Naess–Gaidai method^25,26^ is based on the fact that for most engineering applications, the exceedance probability tail of response processes exhibits regularity, enabling accurate extrapolation up to extreme levels. The mean up-crossing rate as a function of the response level is in general regular in the tail, i.e. for sufficiently high values of $\zeta$. The mean up-crossing rate tail, say for $\zeta \ge \zeta_{0}$, behaves closely like

8 $$\nu_{Z}^{ + } \left( \zeta \right) \approx q{\text{exp}}\left( { - a\left( {\zeta - b} \right)^{c} } \right), \zeta \ge \zeta_{0} > b$$

where $a, b, c, q$ being suitable constants. Thus, as discussed in detail in^25,26^, plotting ${\text{log}}\left| {{\text{log}}\left( {\nu_{Z}^{ + } \left( \zeta \right)/q} \right)} \right|$ versus ${\text{log}}\left( {\zeta - b} \right)$, exhibits an almost perfectly linear tail behaviour. Values of $a, b, c, q$ can be estimated by using optimization suitable procedure, see^25,26^.

Since this paper studies springing response, which typically involves narrow band episodes (clustered local maxima), it is in fact preferable to use ACER (Average Conditional Exceedance Rate) method^27^, since Naess–Gaidai method will somewhat overestimate response prediction due to not accounting for clustering effects. Still, for practical purposes it is shown that Naess–Gaidai method is accurate enough.

## Results

This section presents numerical results for a specific TLP example structure. Wave radiation forces have been determined using WAMIT hydrodynamic software^28^, based on the TLP panel model. The TLP analysed here is of relatively small size, with about 12.3 m column diameter and about 80.2 m wide. Initially the solo heave, roll and pitch responses have been considered, thereafter the latter solo responses have been combined into the TLP corner point total vertical displacement. A single degree of freedom model has been used for each solo response (heave, roll and pitch) separately. For the heave, roll and pitch, the TLP particulars are given in Table 1. For the particular TLP studied here, the roll and pitch moment arm associated with the tether forces is $l$ = 17.42 m. Note that roll and pitch in the present paper refer to rotation around the centre of gravity (CoG) origin at the mean water level.

**Table 1:** TLP particulars.

	Heave	Roll	Pitch
Eigen period $T_{e}$ (s)	4.21	5.43	5.44
Relative damping $\xi$(%)	1.03	2.04	2.06
Total mass $M$ (kg)	$10^{7}$	$-$	$-$
Total mass moment $I$ of inertia (kg $\cdot {\text{m}}^{2}$)	$-$	$4.64 \cdot 10^{9}$	$4.64 \cdot 10^{9}$

Note that Table 1 presents values of mass, moment of inertia etc. calculated for an existing TLP, studied in this paper. Also note that the total mass and the total mass moment of inertia presented in Table 1 include added mass and added moment of inertia, according to WAMIT calculation, see^29^, the latter are weakly dependent on the wave frequency $\omega$ and therefore assumed to be constant.

In the following, the wave direction is assumed to be parallel to the roll axis ($x$-axis), while the $x$−$z$ plane is being a plane of symmetry, making roll excitation and response to be zero. Therefore, only pitch is further discussed. For configuration of TLP and tendons and coordinate system see Fig. 1.

Note that the second order theory is based on the assumption that the heave, roll or pitch motion response QTF, both having the form $\hat{H}_{2} \left( {\omega_{k} , - \omega_{l} } \right) = \hat{L}\left( {\omega_{k} - \omega_{l} } \right)\hat{K}_{2} \left( {\omega_{k} , - \omega_{l} } \right)$, where $\hat{K}_{2} \left( { \cdot , \cdot } \right)$ being the QTF characterizing the wave forces on the TLP, and $\hat{L}\left( \cdot \right)$ is a linear transfer function for the heave, roll or pitch motion of the TLP, that is $\hat{L}\left( \omega \right) = \frac{1}{{M\left( \omega \right)\left[ { - \omega^{2} + 2i\xi \omega_{e} \omega + \omega_{e}^{2} } \right]}}$ where $\omega_{e} = \frac{2\pi }{{T_{e} }}$ being the resonance frequency. Note that $M$ includes added mass and it is assumed to be weakly dependent on the wave frequency $\omega$. The $\hat{K}_{2}$ QTF matrix can be expressed in the same Hermitian form as $\underline{\underline{Q}}$-matrix in Eq. (4)

9 $$\hat{K}_{2} = \left( {\begin{array}{*{20}c} 0 & {\underline{\underline{K}}^{H} } \\ {\underline{\underline{K}} } & 0 \\ \end{array} } \right)$$

with the non-dimensionalized $N \times N$ matrix $\underline{\underline{K}}$ being calculated using WAMIT—the second order diffraction software^28^, for the particular TLP considered in this paper. The discrete wave frequency range, that corresponds to the matrix $\underline{\underline{K}}$ is as follows $\omega_{1} = \frac{2\pi }{{30.0}},.. , \omega_{N} = \frac{2\pi }{{4.0}}$(rad/s) with $N = 40$.

There is the following specific requirement that must be observed, in order to get an accurate representation of the response process. Namely, since the heave damping coefficient is only about 1%, the resonance peak of the linear transfer function is narrow. In order to estimate the TLP dynamics correctly, the wave frequency resolution must have a sufficient number of discrete frequency values over the latter resonance peak.

**Figure 2:**
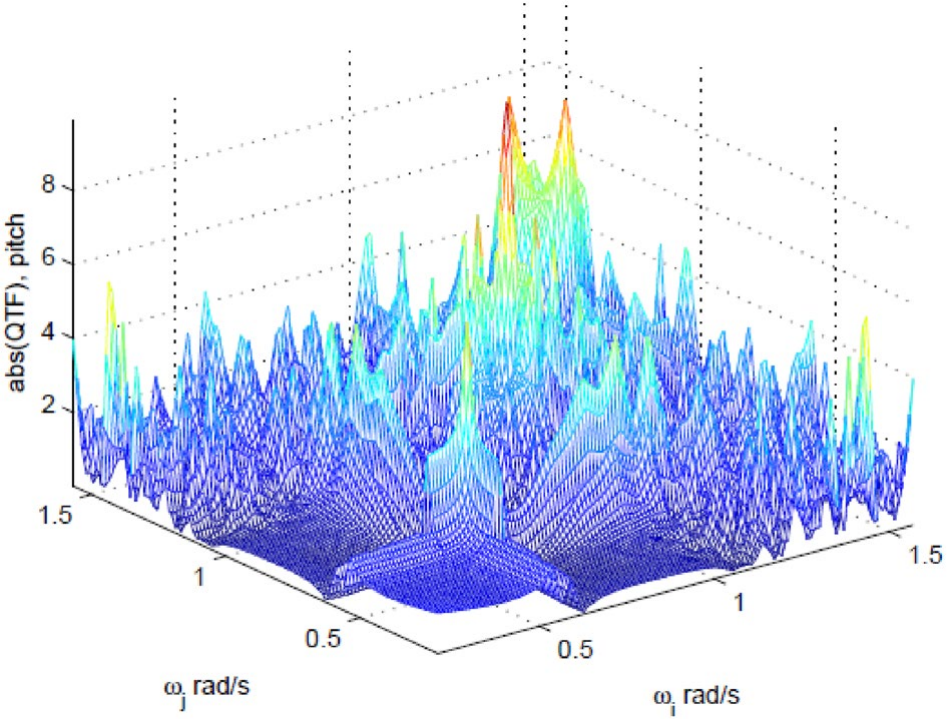
Non-dimensional pitch QTF absolute value, using MATLAB, www.mathworks.com.

Figure 2 presents the non-dimensional pitch moment QTF, computed within WAMIT user manual recommendation^28^. The software package MATLAB^30^ was used to solve the two-dimensional matrix interpolation problem, as well as the corresponding eigenvalue problem. The interpolated wave frequencies $\overline{\omega }_{k}$,$\overline{\omega }_{1} < \overline{\omega }_{k} < \overline{\omega }_{N}$ have been chosen to be equidistant in the range $\left[ { \overline{\omega }_{1} , \overline{\omega }_{N} } \right]$, with $\overline{\omega }_{1} = 0.26$*;* $\overline{\omega }_{N} = 1.26$ rad/s.

Figure 2 presents the absolute value of the pitch QTF. The random stationary sea state is given by the JONSWAP PSD, see^4^

10 $$S_{\eta } \left( \omega \right) = \frac{{\alpha g^{2} }}{{\omega^{5} }}{\text{exp}}\left\{ { - \frac{5}{4}\left( {\frac{{\omega_{p} }}{\omega }} \right)^{4} + {\text{ln}}\gamma \cdot {\text{exp}}\left[ { - \frac{1}{{2\sigma^{2} }}\left( {\frac{\omega }{{\omega_{p} }} - 1} \right)^{2} } \right]} \right\}$$

with $g = 9.81$ ms^−2^, and $\omega_{p}$ being the wave peak frequency in rad/s and where $\gamma$ and $\sigma$ being parameters affecting the spectral shape; $\sigma = 0.07$ when $\omega \le \omega_{p}$, and $\sigma = 0.09$ when $\omega > \omega_{p}$. The peakness parameter $\gamma$ has been chosen to be equal to 3.3. The parameter $\alpha$ has been estimated from the following empirical equation

11 $$\alpha = 5.06\left( {\frac{{H_{s} }}{{T_{p}^{2} }}} \right)^{2} \left( {1 - 0.287{\text{ln}}\gamma } \right)$$

with $H_{s}$ being the significant wave height and $T_{p} = \frac{2\pi }{{\omega_{p} }}$ being the spectral peak wave period. For the subsequent discussion, two different sea states have been chosen:

1. $H_{s} = 4.3$ m and $T_{p} = 9.5$ sec (referred to as the “moderate sea state”)
2. $H_{s} = 7.1$ m and $T_{p} = 16.5$ sec (referred to as the “severe sea state”)

The natural frequency in heave has been estimated as $\omega_{e} = 1.51$ rad/sec, and in roll and pitch as $\omega_{e} = 1.16$ rad/sec, both latter frequencies are being above the main wave energy range for the current choices of $T_{p}$. Although the wave energy around the resonance frequency is quite small, it cannot be neglected, moreover in the springing analysis the linear high frequency wave loads should be included as well. The latter observation is more valid for pitch than heave, since the pitch resonance period is about 30% larger than for heave. Since the second order non-linear term in Volterra expansion is often the most important one, it is therefore critical to accurately capture relevant characteristics of the resonant heave and pitch responses.

For the moderate sea state with $H_{s}$ = 4.3 m and $T_{p}$ = 9.5 s, heave force standard deviation was found at about $5.0 \cdot 10^{5}$ N, with 96% contribution coming from the first order linear force component. For severe sea state with $H_{s}$ = 7 m and $T_{p}$  = 16.5 s, heave force standard deviation was found about $10.1 \cdot 10^{5}$ N, with about 90% contribution coming from the first order force component. The force for the heave calculation is given in Newton (N), and the force moment for pitch calculation is given in Nm.

Similarly for the pitch, the moderate sea state pitch moment standard deviation was found about $1.43 \cdot 10^{7}$ Nm, with only minor contributions from the quadratic sum frequency part. For the severe sea state the pitch moment variance was found about $2.24 \cdot 10^{7}$ Nm, with negligible contribution from the quadratic force moment part.

The pitch induced response has been expressed in cm, by means of multiplication of the pitch response by the pitch arm $l$. Non-dimensional response then was obtained by dividing the response with its standard deviation. For the moderate sea state, heave response standard deviation was found about 3.81 cm, with about 75% contribution is from the linear part. For severe sea state heave response standard deviation was found about 6.92 cm, with about 60% contribution is from the linear part. Similarly, for the pitch, the moderate sea state pitch response standard deviation was found about 9.1 cm, with 98% contribution from the linear first order response. For the severe sea state, the pitch response standard deviation was found about 9.68 cm, with about 90% contribution from the linear component.

The latter contribution numbers clearly show how the resonant heave and pitch responses are becoming more significant.

In this paper heave and pitch have been assumed to be decoupled, because of their small magnitudes. Then the linearized vertical corner can be easily estimated as $Z\left( t \right) = Z_{{{\text{heave}}}} \left( t \right) + lZ_{{{\text{pitch}}}} \left( t \right)$. It is observed that the total response standard deviation is almost equal for moderate and severe sea states. More specifically, for the moderate sea state the total response standard deviation was found about 9.8 cm, with 95% due to the linear component. For the severe sea state, the total response standard deviation was found about 12.6 cm, with about 75% due to the linear component. As to be expected, for severe sea nonlinear effect has more relative significance, while for moderate sea linear behaviour is dominating.

While solving the eigenvalue problem given by Eq. (2), the response expression Eq. (1) can be obtained. Note that the above mentioned calculations have to be carried out for each of three responses separately, namely for heave, pitch and the combined corner displacement. Eigenvalues $\mu_{j}$ in Eq. (1), have been sorted in descending order $\mu_{1} > \mu_{2} > \mu_{j}$. Eigenvalues such that ${\text{abs}}\left( {\mu_{j} } \right) < 0.01\mu_{1 }$ are set to zero, meaning that the tolerance level is set to 1%. The latter approximation significantly reduced the number of terms in Eq. (1), as well as significantly accelerated calculation of the mean up-crossing rate, while saving CPU time and not compromising accuracy. As mentioned before, this paper studied unidirectional seas. However, the proposed approach is equally valid for multidirectional seas, leading to a larger eigenvalue problem. Note that, when crossing levels of interest are large, the CPU time needed for exact integration drops rapidly, as compared to the direct Monte Carlo (MC) simulation^1–12^. Since extreme up-crossing rates are quite important ones, this is a practical advantage for the exact saddle point integration method, as compared to the direct MC method.

**Figure 3:**
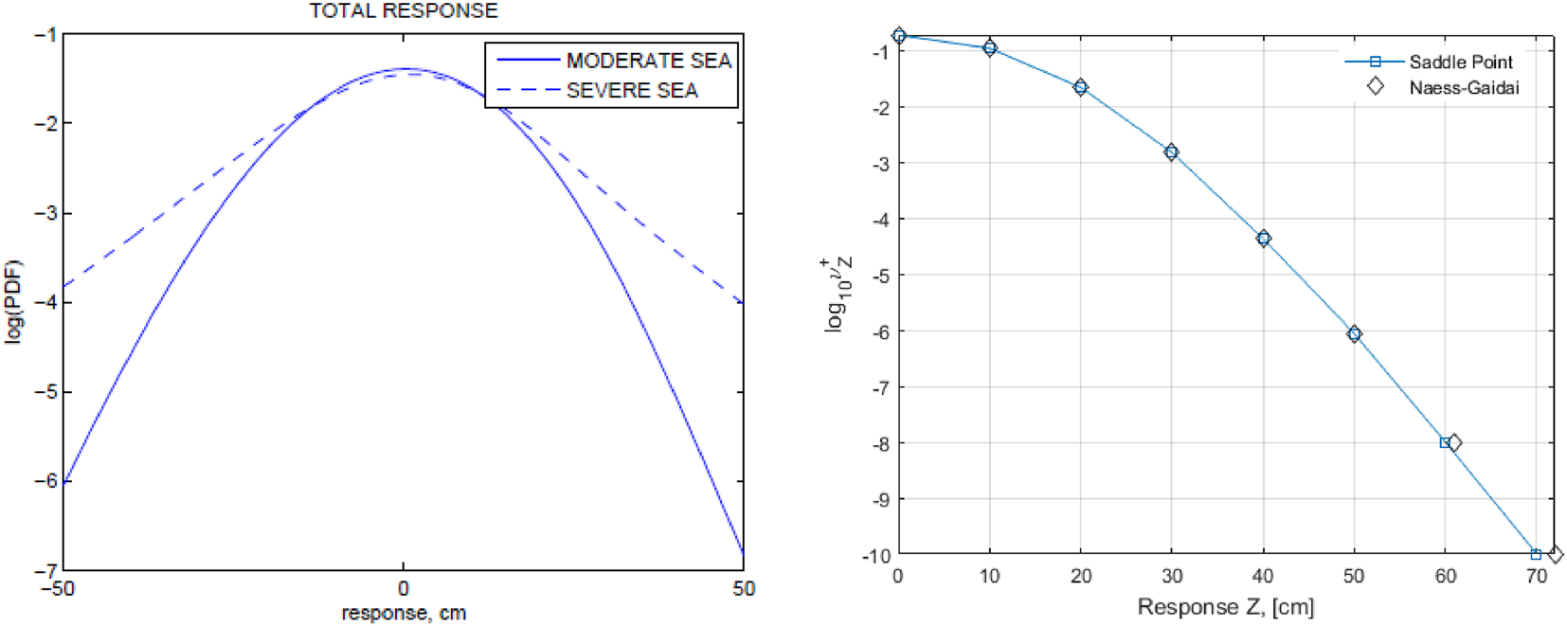
Left: Comparative decimal log PDF of the total response (cm), moderate sea (–) and severe sea (- -). Right: Moderate sea, response mean up-crossing rate comparison on the decimal log scale.

Figure 3 left presents comparative decimal log PDF of the total response (cm), moderate sea and severe sea. As was expected, it is seen from Fig. 3 left that severe sea yields much higher response levels, given the same probability levels as moderate sea. The proposed method is equally efficient for either sea state or their long term combination.

Figure 3 right presents mean up-crossing rate comparison between saddle point (exact) and Naess–Gaidai (extrapolated) on the decimal log scale. Since Naess–Gaidai is a Monte Carlo (MC) based method^25^, it relies on estimated MC crossing rate values up to a certain response level ${\zeta }_{0}$. In this paper, however, authors have chosen to use exact mean up-crossing rate data (obtained by saddle point method), and feed it to Naess–Gaidai extrapolation, in order to see accuracy of prediction for extreme response levels with low probability of up-crossing.

Note that Naess–Gaidai extrapolation in Fig. 3 right was done starting from response level ${\zeta }_{0}=50$ cm (thus using exact data above level ${\zeta }_{0}$ as a base for extrapolation), then extrapolation was done 4 decimal orders of magnitude down, yielding 72 cm response prediction versus exact 70 cm. The latter is about 3% deviation on the response level scale, which can be considered as excellent for practical applications. Alternatively, Naess–Gaidai extrapolation could have been done based on Monte Carlo simulation results, if the latter would be generated. Note that the mean up-crossing is measured in 1/sec and therefore the return period $T$ of interest will be the inverse, so that decimal logarithmic level of −8 on the vertical axis in Fig. 3 right roughly corresponds to $T=1$ year.

It is important to note that for extreme response estimation, the more accurate calculation methods should be used, while for fatigue calculations of TLP tendons, one may in fact use the simplest approach for the calculation of the crossing rates.

## Discussion

In this paper authors have investigated a general numerical method (exact) for calculating the mean level up-crossing rates of a second order stochastic Volterra series, by highlighting combined heave and pitch response of a TLP in random seas. The method has been successfully implemented, and numerical results have been presented.

Exact results were compared to approximate Naess–Gaidai method predictions. Naess–Gaidai method is the Monte Carlo based extrapolation technique, that enables accurate prediction of mean up-crossing rate tail. The latter appears to provide estimates of crossing rates that are quite accurate for high and extreme response levels.

An advantage of using Naess–Gaidai method is that it enables fast and accurate extrapolation down to extreme response levels with low probability of up-crossing.

The response levels obtained by de-clustering technique are up to 10% lower than those predicted by the mean up-crossing rate methods. The latter is certainly a useful correction that may reduce overall cost to build the reliable TLP structure.

Since this paper is focused mainly on post-processing of direct Monte Carlo (MC) simulation results, there is no focus on the simulation length and CPU time, since there is no comparison to a direct full MC simulation (as in e.g.^21,31–39^), but rather a comparison with CPU time efficient analytical solution.

Finally, the presented methodology has following important advantages:

- Any kind of TLP response or loading data can be analysed: either measured or numerically simulated. The only assumption is ergodicity.
- Conventional methods like e.g. Gumbel in practice require equally probable maxima, therefore they do not account for environmental probabilities, while Naess–Gaidai method is well suitable to account for environmental distributions.
- Unlike asymptotic methods (Gumbel, Weibull, Pareto etc.) the presented method may be regarded as pre-asymptotic, which means that the same data set can be analysed more accurately and efficiently.

Higher order TLP springing and ringing response components can be easily accommodated by Monte Carlo simulation, however analytical (saddle point) solution can’t be produced, therefore both advocated methods can’t be cross-validated. This paper is primarily focused on statistical methodology, not that much on structural and hydrodynamic technical details.

## Data Availability

The datasets analysed during the current study are available on request from corresponding author Dr. Jingxiang Xu, jxxu@shou.edu.cn.
